# Stochastic and Arbitrarily Generated Input Patterns to the Mushroom Bodies Can Serve as Conditioned Stimuli in *Drosophila*

**DOI:** 10.3389/fphys.2020.00053

**Published:** 2020-02-11

**Authors:** Carmina Carelia Warth Pérez Arias, Patrizia Frosch, André Fiala, Thomas D. Riemensperger

**Affiliations:** Department of Molecular Neurobiology of Behavior, Johann-Friedrich-Blumenbach-Institute of Zoology and Anthropology, University of Göttingen, Göttingen, Germany

**Keywords:** *Drosophila melanogaster*, olfactory projection neurons, mushroom body, antennal lobe, learning and memory, thermogenetics, olfaction

## Abstract

Single neurons in the brains of insects often have individual genetic identities and can be unambiguously identified between animals. The overall neuronal connectivity is also genetically determined and hard-wired to a large degree. Experience-dependent structural and functional plasticity is believed to be superimposed onto this more-or-less fixed connectome. However, in *Drosophila melanogaster*, it has been shown that the connectivity between the olfactory projection neurons (OPNs) and Kenyon cells, the intrinsic neurons of the mushroom body, is highly stochastic and idiosyncratic between individuals. Ensembles of distinctly and sparsely activated Kenyon cells represent information about the identity of the olfactory input, and behavioral relevance can be assigned to this representation in the course of associative olfactory learning. Previously, we showed that in the absence of any direct sensory input, artificially and stochastically activated groups of Kenyon cells could be trained to encode aversive cues when their activation coincided with aversive stimuli. Here, we have tested the hypothesis that the mushroom body can learn any stochastic neuronal input pattern as behaviorally relevant, independent of its exact origin. We show that fruit flies can learn thermogenetically generated, stochastic activity patterns of OPNs as conditioned stimuli, irrespective of glomerular identity, the innate valence that the projection neurons carry, or inter-hemispheric symmetry.

## Introduction

**FIGURE 1:**
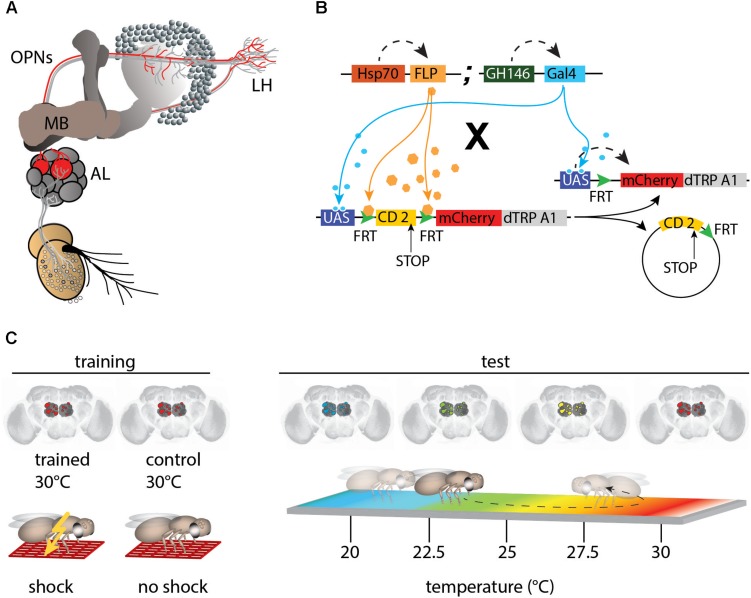
mCherry-dTRPA1 expression in random subsets of olfactory projection neurons (OPNs). **(A)** Illustration of the olfactory pathway in *Drosophila*. Odors are detected by olfactory receptor neurons (ORNs) that project to the antennal lobes (AL). Olfactory information is conveyed by OPNs from the AL to the calyx of the mushroom bodies (MBs) and the lateral horns (LH). **(B)** Random expression of mCherry-dTRPA1: Flies carrying GH146-Gal4 and a flippase under Hsp-70 control were crossed with flies that carried a UAS-coupled FRT-flanked CD2 (stop) cassette followed by the mCherry-dTRPA1 DNA construct. Heat shock-mediated activation of the flippase causes a random excision of the FRT-cassette and transcription of mCherry-dTRPA1. **(C)** Schematic depiction of the training protocol and behavioral read-out. The OPNs were thermogenetically activated for 2 min. One group of flies was simultaneously exposed to electric shocks (“Training”) while a second group of flies was treated similarly but did not receive electric shocks and served as controls. Subsequently, the flies were individually transferred to a temperature gradient arena (“Test”) where they could freely distribute according to their temperature preference. Through movements along the temperature gradient, the flies triggered or avoided thermogenetic depolarization of the OPNs expressing mCherry-dTRPA1.

*Drosophila melanogaster* is a model organism, widely used in studies of the neuronal basis of behavior. This is not only because of the wealth of elaborate genetic tools available to allow specific neurons can be genetically targeted and manipulated (Venken et al., 2011), but also to the highly individually stereotypic and genetically determined connectivity between neurons that facilitate the analytical dissection of neuronal circuits (Meinertzhagen and Lee, 2012). The stereotypic connectivity in *Drosophila* contrasts with that in the vertebrate brain, where neurons usually cannot be identified at the level of individual cells and compared across animals. One well-studied example of highly stereotypic connectivity is the olfactory system of the fruit fly (Grabe and Sachse, 2018). Olfactory sensory neurons express olfactory receptors that have evolved to detect behaviorally relevant odorants. Sensory neurons that express the same receptors project into the same glomeruli in the antennal lobes that also have stereotypic anatomical locations at the inter-individual and inter-hemispheric levels (Couto et al., 2005; Fishilevich and Vosshall, 2005). As a result, stereotypic chemotopic maps of odor representations can be detected across individuals (Rodrigues and Buchner, 1984; Fiala et al., 2002). Olfactory projection neurons (OPNs), the second-order olfactory neurons of the insect brain receive input from the glomeruli of the antennal lobes and target the lateral horn and the calyx of the mushroom body (schematically depicted in Figure 1A). Their individual identities and anatomical projections to the target brain regions are also relatively constant across individuals (Marin et al., 2002; Wong et al., 2002; Tanaka et al., 2004; Jeanne et al., 2018). Even at the level of gene expression, individual OPN types can be unambiguously distinguished, highlighting their genetic individualities (Li et al., 2017). This connectivity also leads to chemotopic maps in those higher-order brain regions targeted by OPNs (Fiala et al., 2002; Jefferis et al., 2007). This deterministic, hard-wired connectivity, together with specific sensory receptors, has led to the idea of multiple neuronal “labeled lines,” wherein each olfactory input stimulates a route of connections that ultimately evoke an appropriate behavioral response, such as the avoidance of harmful substances (Suh et al., 2007; Stensmyr et al., 2012), egg-laying on odorous substrates (Dweck et al., 2015), or pheromone-induced courtship behavior (van der Goes van Naters and Carlson, 2007; Datta et al., 2008).

However, at least one exception from the rule of genetically determined, hard-wired connectivity exists in the *Drosophila* brain olfactory system. The ∼2000 intrinsic neurons of the mushroom body (Kenyon cells) per hemisphere (Aso et al., 2009) receive stochastic input from ∼150 OPNs, and the dendrites of each Kenyon cell receive input from several OPNs (Caron et al., 2013). Substantial evidence has accumulated showing that the process of associative olfactory learning is localized to the mushroom bodies (Heisenberg et al., 1985; de Belle and Heisenberg, 1994; Connolly et al., 1996; Zars et al., 2000; Dubnau et al., 2001; McGuire et al., 2001; Qin et al., 2012). In particular, to the lobes as the main output structures (Aso et al., 2014a). During the learning regime, an odor is presented as a conditioned stimulus in temporal coincidence with a rewarding or punishing unconditioned stimulus, such as sugar or an electric shock (Tempel et al., 1983; Tully and Quinn, 1985). Each odor evokes the activity of a distinct, sparsely distributed ensemble of Kenyon cells (Honegger et al., 2011). The axons of the Kenyon cells are compartmentalized such that distinct populations of dopaminergic neurons, which signal rewarding or punishing unconditioned stimuli, innervate distinct partitions (Aso et al., 2014b). It has long been accepted that the coincidental release of dopamine onto Kenyon cell axons and the odor-evoked calcium influx therein causes presynaptic modification of transmitter release onto a small number of mushroom body output neurons that instruct the behavior of the animal (Heisenberg, 2003; Aso et al., 2014a) and that are, again, highly anatomically stereotypic at the inter-hemispheric and inter-individual levels (Aso et al., 2014b).

To summarize, data collected to date suggest that the large number of Kenyon cells organized in parallel are not identifiable at the individual-neuron level, and show random connectivity with OPNs (Caron et al., 2013). In addition, distinct stimuli evoke non-stereotypic, idiosyncratic activity patterns across Kenyon cells (Murthy et al., 2008; Gruntman and Turner, 2013). It has been proposed that the stochastic nature of OPN-to-Kenyon cell connectivity and the resulting stochastic response patterns of Kenyon cells could reflect the evolutionary unpredictability of stimuli to be learned. In this sense, the stimulus-activated ensembles of Kenyon cells do not encode odors, visual images, or tastes. Rather, they encode arbitrary patterns to which value(s) can be assigned through learning, and which can ultimately instruct behavior. They are arbitrary in the sense that no genetically determined pattern of Kenyon cell activity or circuit diagram carries any behaviorally relevant information about the odor or any other stimulus. In a previous study, we showed that artificially and stochastically activated groups of Kenyon cells, in coincidence with a punishing electric shock, can be learned as aversive cue without direct sensory input (Vasmer et al., 2014). By modifying their behavior, trained animals avoided reactivation of those Kenyon cell ensembles whose activities were associated with the punishment. This finding suggests that the overall Kenyon cell array can learn any neuronal input pattern to be avoided, independent of the nature of the actual sensory stimulus and inter-hemispheric symmetry. Here, we tested this hypothesis. Our results indicate that fruit flies can indeed learn to avoid stochastic, thermogenetically generated OPN activity patterns as conditioned stimuli.

## Materials and Methods

### *Drosophila* Strains

Fly stocks were raised on standard cornmeal-agar medium at 18°C, 60% relative humidity, and under a 12 h light–dark cycle. Flies were generated as described by Vasmer et al. (2014). A Hsp70-FLP insertion on the X-chromosome (provided by G. Struhl) was combined with the GH146-Gal4 (Stocker et al., 1997) insertion on the second chromosome to generate a strain homozygous for both P-element inserts. These flies were crossed with a strain carrying the UAS-FRT-CD2(Stop)-FRT-mCherry-dTRPA1 (Vasmer et al., 2014; Pooryasin and Fiala, 2015) construct with the insertion balanced over CyO. Female F1 offspring younger than 2 days were anesthetized using CO_2_ and transferred to a fresh food vial. Unless otherwise indicated, flies were incubated at 30°C for 4 h to induce FLP-mediated expression. Figure 1B illustrates how this causes stochastic heat-shock-induced expression of mCherry-dTRPA1 in neurons determined by the Gal4 line.

### Behavioral Analysis

Flies were trained as described by Vasmer et al. (2014), schematically illustrated in Figure 1C. Female flies aged 4–6 days old were transferred into pre-warmed (30°C) tubes covered on the inside with an electrifiable copper grid. Training was performed in an illuminated incubator at 30°C and at an air humidity of ∼60%. Animals were kept in these tubes for 2 min, during which time 24 90 V DC electric shocks of 1.25 s each in duration were applied, separated by 3.75 s breaks, for a total shock interval of 5 s. Control animals were treated in a similar manner; that is, they were exposed to a temperature of 30°C in the same tubes but did not receive the electric shocks. Subsequently, the flies were transferred to a heat-gradient chamber (schematically depicted in Figure 1C, right) that consisted of an aluminum block with eight walking tracks (275 mm length × 5 mm width × 4 mm height) covered with a Plexiglas lid. The entire apparatus was kept in an incubator under a constant white light, and at a temperature of 16°C and ∼65% humidity, producing a linear and stable temperature gradient over the length of the arena ranging from 18 to 35°C. Individual flies were transferred without anesthesia into the walking tracks through small holes in the lid and were permitted to move freely for 20 min. Locomotion was monitored from above using a high-definition video camera (Panasonic HC-V500). Flies were tracked using the Noldus Ethovision XT 8.5 software (Wageningen) to generate data used in the analysis. The temperature preference of each fly over the observation period of 20 min was determined in 5 min time bins as the time spent above or below 24°C. The flies were anesthetized immediately after the behavioral experiments and their brains were dissected. Localization of mCherry-dTRPA1 expression was determined using immunohistochemistry.

### Immunohistochemistry

Brains were dissected in ice-cold Ringer’s solution containing 5 mM Hepes (pH 7.3), 130 mM NaCl, 5 mM KCl, 2 mM MgCl_2_, 2 mM CaCl_2_, and 36 mM sucrose (Estes et al., 1996) and fixed for 2 h on ice in 4% paraformaldehyde dissolved in phosphate-buffered saline (PBS). Subsequently, brains were washed three times for 20 min each in PBS containing 0.6% Triton X-100 (PBST), then incubated in PBST containing 2% bovine serum albumin (block solution) for 2 h. Afterward, the brains were incubated overnight at 4°C in block solution containing mouse anti-nc82 antibody against Bruchpilot (provided by Erich Buchner) (Wagh et al., 2006) diluted 1:10. Brains were again washed three times for 20 min each in PBST and incubated for at least 4 h with goat anti-mouse 1:300 conjugated with Alexa Fluor 488-conjugated goat anti-mouse (Invitrogen). Brains were washed three times in PBST for 20 min each, washed in PBS overnight at 4°C, and embedded in Vectashield (Vector Laboratories). Images were acquired using a confocal laser scan microscope (Leica SP8) equipped with hybrid detectors and analyzed using *ImageJ*. The antennal lobe glomeruli were determined using anti-bruchpilot (anti-*Brp*) immunoreactivity and mCherry-dTRPA1 expression was characterized. The antennal lobes of both hemispheres were examined when possible.

### Statistical Analysis

The symmetry index was defined as the ratio between symmetrically innervated glomeruli by OPNs expressing dTRPA1-mCherry and the total number of innervated glomeruli by mCherry-dTRPA1 expressing OPNs. All statistical tests were conducted using *GraphPad Prism7* and *OriginPro* software. A Kolmogorov–Smirnov test was used to test for a normal distribution of data. Normally distributed data were analyzed using one-way ANOVA followed by Bonferroni *post hoc* tests for multiple pairwise comparisons. Non-normally distributed data were analyzed using the Kruskal–Wallis test followed by Dunn’s *post hoc* tests for multiple pairwise comparisons. For correlation analyses, Spearman correlations were calculated. For testing for randomness of dTRPA1-mCherry expression in glomeruli a Runs test (Wald–Wolfowitz test) was conducted for all flies and for each glomerulus and each brain hemisphere independently.

## Results

### Expression of mCherry-Tagged dTRPA1 in Stochastic Ensembles of OPNs

To obtain expression of mCherry-dTRPA1 in stochastic subsets of OPNs (Figure 1B), flies carrying the DNA construct of a mCherry-tagged thermo-inducible cation-channel dTRPA1 (Vasmer et al., 2014; Pooryasin and Fiala, 2015) were crossed with flies carrying a flippase under control of the heat shock promoter Hsp-70 (Basler and Struhl, 1994) together with the GH146-Gal4 driver line (Stocker et al., 1997; Figure 1B). The groups of neurons that express dTRPA1 can be artificially depolarized by raising the temperature above ∼25°C (Hamada et al., 2008; Tang et al., 2013; Vasmer et al., 2014; Pooryasin and Fiala, 2015). dTRPA1 induces a relatively sharp increase in excitation in neurons expressing it, with a peak at ∼32°C (Hamada et al., 2008; Tang et al., 2013). The flippase-mediated mCherry-dTRPA1 expression is induced by heat shock within the first 2 days after hatching. To test whether the animals can associate the activity of stochastic sets of OPNs with a punishing electric shock, OPNs expressing mCherry-dTRPA1 were thermogenetically activated at 30°C and this activation was temporally paired with 2 min of electric shocks of 90 V. The shocks were 1.25 s in duration and separated by 3.75 s intervals, as described (Vasmer et al., 2014). Control animals of the same genotype were treated in the same manner but did not receive the electric shocks (Figure 1C). In a typical aversive olfactory conditioning procedure, the animals learn to avoid the odor that has been temporally paired with the punishment (Tully and Quinn, 1985). In our experiment, the stochastically distributed activity of OPNs did not reflect any real odor representation that could be tested for. To bypass any olfactory input also in the memory test phase, directly after the training procedure the animals were individually transferred to a test chamber in which they could walk freely along a temperature gradient ranging from 18 to 35°C (Figure 1C right). The movement of each animal was monitored and temperature preference during the first 5 min was used as the memory readout. The mCherry-dTRPA1 channel starts opening at ∼25°C (Vasmer et al., 2014); that is, within the preferred temperature range of naïve fruit flies (Sayeed and Benzer, 1996; Hamada et al., 2008). Therefore, the relative amount of time the animals spent >24°C, i.e., at a temperature that is still within the range of their innate temperature preference but just below the onset of dTRPA1-mediated excitation, after training was used to determine whether the animals approached or avoided re-activation of the population of OPNs expressing mCherry-dTRPA1.

### Thermogenetic Induction of Associative Learning

**FIGURE 2:**
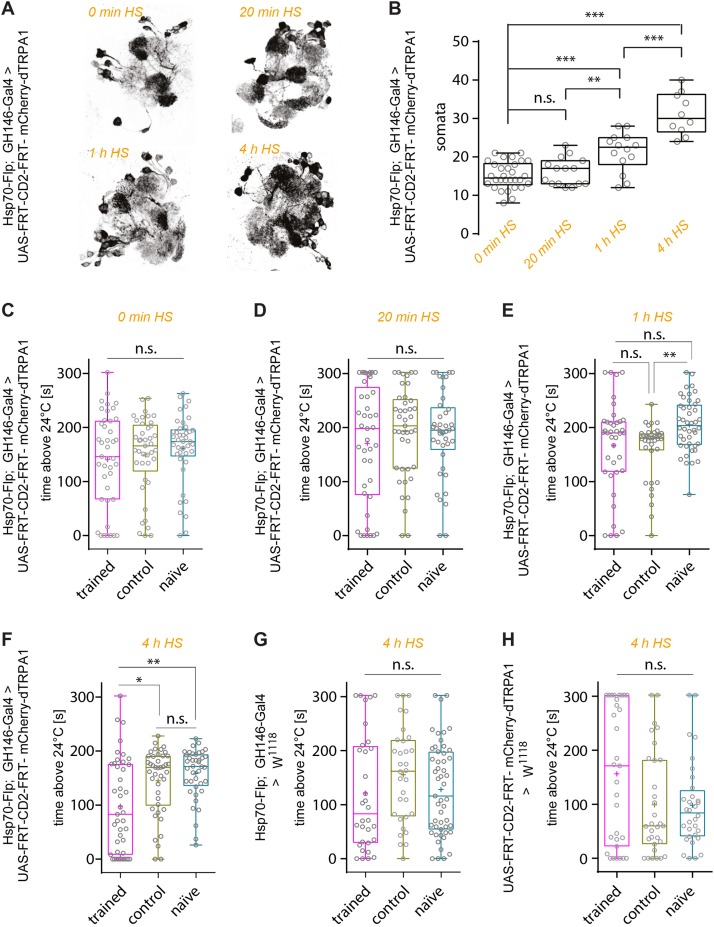
Thermogenetic induction of associative learning. **(A)** Expression of mCherry-dTRPA1 in random ensembles of OPNs in the AL resulting from a 0 min, 20 min, 1 h, and 4 h heat exposure during development. **(B)** Prolonged exposure to heat during development causes mCherry-dTRPA1 expression in an increased number of OPNs expressing mCherry-dTRPA1. **(C–H)** Average time the flies spent >24°C in four different groups of animals expressing mCherry-dTRPA1 in random ensembles of OPNs. Flies that were treated at 30°C with electric shocks (“shock”) were compared to flies that were treated similarly but did not receive electric shocks (“control”) and to “naïve” flies that did not receive any pre-treatment. The four groups differed in the duration of the heat shock-mediated induction of gene expression and the average number of OPNs expressing mCherry-dTRPA1. No significant differences between the three groups were observed in flies that did not receive any heat shock **(C)** or a 20 min exposure **(D)**. In animals that received a 1 h induction, only naïve individuals showed significantly higher activity than control animals. However, the test group did not differ significantly from control animals. Animals that received a 4 h induction **(F)** of gene expression showed a significant reduction in the mean time spent >24°C during the trial compared to both control and “naïve” flies. Genetic control strains that carried the UAS construct only **(G)** or the flippase and the Gal4 constructs **(H)** did not show differences between the three groups. n.s., *p* > 0.05; ^∗^*p* < 0.05; ^∗∗^*p* < 0.01; ^∗∗∗^*p* < 0.001. Box plots indicate medians, interquartile ranges, and minimum and maximum values; means are indicated by crosses.

First, the effect of heat shock duration during development on the number of mCherry*-*dTRPA1 expressing OPNs was examined: The longer and more often the heat shock was, the more neurons should express mCherry-dTRPA1. This was the case as an increase in heat exposure time after hatching gradually increased the number of mCherry*-*dTRPA1 expressing OPNs, as determined by quantifying mCherry-labeled somata (Figures 2A,B and Table 1). We then asked whether flies can associate activation of OPNs with a punishment, and whether this association depends on the number of activated OPNs. Therefore, four groups of flies were tested, namely, flies that did not receive any heat-induction of mCherry-dTRPA1 expression during development and thus only expressed the DNA construct in small populations of random subsets of OPNs, and flies that received a 20 min, 1 h, or 4 h heat induction of mCherry-dTRPA1 in OPNs. All groups were subjected to electric shocks simultaneous to the thermogenetic induction of neuronal activity. These groups were compared with control flies that did not receive electric shocks and with “naïve” flies that were not exposed to either increased temperature or electric shocks. Flies without heat-induction expressed mCherry-dTRPA1 in only 15.17 ± 0.64 (mean ± SEM; Figure 2B). OPNs and did not show any significant changes in temperature preference compared with control or naïve flies (Figure 2C and Table 2). Similarly, we did not find a change in the preferred temperature of trained flies that received a 20 min or 1 h induction of expression (Figures 2D,E and Table 2). However, flies expressing mCherry-dTRPA1 in random subsets of 31.1 ± 1.72 (mean ± SEM; Figure 2F) OPNs after a 4 h induction of expression during development showed a significant shift toward lower temperatures when treated with punitive electric shocks simultaneously with OPN activation (Figure 2F and Table 2). Genetic controls, i.e., the heterozygous UAS > CD2 > mCherry-dTRPA1 strain and the heterozygous Hsp-70-FLP; GH146-Gal4 strain, that received the same duration of heat shock during development and training did not show any difference in temperature preference after training compared with control and naïve animals (Figures 2G,H and Table 2). This finding indicates that the animals actively prevented reactivation of the OPNs if the activity of a sufficient number of OPNs was paired with a punishment, i.e., through associative learning. However, this learned avoidance was restricted to the first 5 min within the observation time period. At later time points the temperature avoidance was not different between test and control groups (Supplementary Figure 1).

**TABLE 1:** Prolonged exposure to heat during development causes mCherry-dTRPA1 expression in an increased number of OPNs expressing mCherry-dTRPA1.

	**Heat shock duration**	**Somata/hemisphere**	***SD***	***n***	**Bonferroni corr. one-way ANOVA**	**Somata/difference between hemispheres**	***SD***	**Bonferroni corr. one-way ANOVA**
	
Hsp70-Flp; 146-Gal4 >	0 min	15.17	2.749	15		3.267	2.987	*p* > 0.05
USA-FRT-CD2-FRT-	20 min	16.38	3.303	8	vs. 0 min, *p* > 0.05	2.857	1.864	*p* > 0.05
mCherry-dTRPA1	1 h	21.29	4.05	7	vs. 0 min, *p* = 0.0027	5.429	3.309	*p* > 0.05
	4 h	31.1	4.436	5	vs. 0 min, *p* < 0.0001	5.4	4.159	*p* > 0.05
					vs. 20 min, *p* < 0.0001		
					vs. 1 h, *p* = 0.0002		
								

**TABLE 2:** Prolonged induction of mCherry-dTRPA1 expression in OPNs significantly reduces the mean time flies > 24°C during the test situation compared to both control and “naïve” flies.

	Heat shock duration	Exp. group	*n*	Mean time [s] >24°C	Lower 95% Cl of mean	Upper 95% Cl of mean	Kruskal–Wallis test with Dunn’s multiple comparsion			Figures
Hsp70-Flp; GH146-Gal4 >	0 min	Trained	40	144.3	116.8	171.7	vs. control	*p* > 0.999	n.s.	2C
UAS-FRT-CD2-FRT-		Control	40	153.3	131.3	175.2	vs. naive	*p* > 0.999	n.s.	
mCherry-dTRPA1		Naive	40	165.6	146.3	184.8	vs. trained	*p* = 0.8064	n.s.	
	20 min	Trained	41	170.8	136.1	205.4	vs. control	*p* > 0.999	n.s.	2D
		Control	41	192.4	166.0	218.8	vs. naive	*p* > 0.999	n.s.	
		Naive	41	188.7	163.6	213.7	vs. trained	*p* > 0.999	n.s.	
	1 h	Trained	39	166.7	139.3	194.2	vs. control	*p* = 0.5091	n.s.	2E
		Control	36	159.8	141.4	178.2	vs. naive	*p* = 0.0021	**	
		Naive	44	205.2	190.2	220.1	vs. trained	*p* = 0.1296	n.s.	
	4 h	Trained	43	97.86	71.12	124.6	vs. control	*p* = 0.0189	*	2F
		Control	42	144.9	126.0	163.7	vs. naive	*p* > 0.999	n.s.	
		Naive	40	158.7	143.6	173.7	vs. trained	*p* = 0.001	**	
Hsp70-Flp; GH146-Gal4	4 h	Trained	31	156.3	109.7	203.0	vs. control	*p* = 0.3283	n.s.	2G
		Control	31	98.94	63.72	134.1	vs. naive	*p* = 0.7226	n.s.	
		Naive	32	98.03	69.29	126.8	vs. trained	*p* > 0.999	n.s.	
UAS-FRT-CD2-FRT-	4 h	Trained	32	121.3	82.95	159.6	vs. control	*p* = 0.3072	n.s.	2H
mCherry-dTRPA1		Control	32	155.3	123.5	187.1	vs. naive	*p* = 0.6088	n.s.	
		Naive	52	128.2	104.3	152.0	vs. trained	*p* > 0.999	n.s.	

n.s., *p* > 0.05; *p < 0.05; **p < 0.01; ***p < 0.001.

### No Correlation Between Activated Number of OPNs and Memory Expression

**FIGURE 3:**
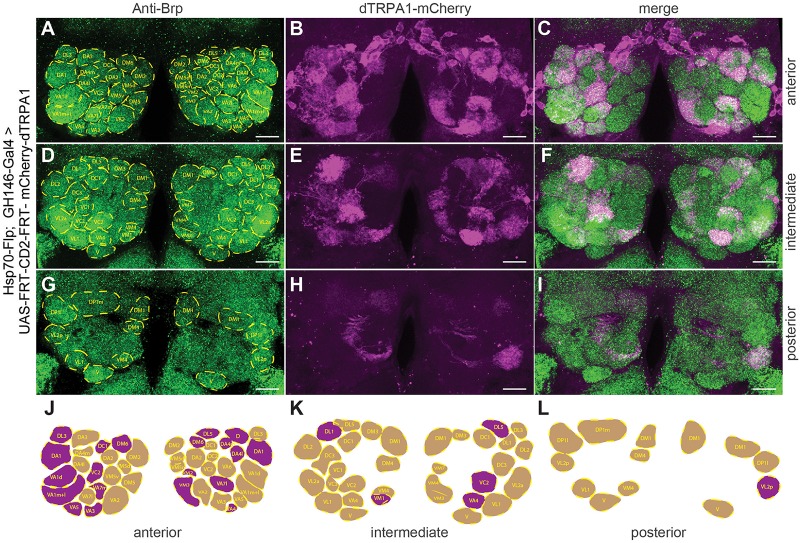
Stochastic OPN expression pattern of mCherry-dTRPA1 in the antennal lobes of one exemplary fly. **(A)** Immunostaining against the pre-synaptic protein Brp (green, left, lower panel) for identification of glomeruli. **(B,E,H)** mCherry-dTRPA1 expression in OPNs in anterior **(A)**, intermediate **(D)**, and posterior **(G)** optical sections across the antennal lobes and the according merged images **(C,F,I)**. Scale bar: 10 μm. **(J–L)** Schematic illustration of olfactory glomeruli expressing mCherry-dTRPA1 (magenta) and remaining glomeruli (beige).

**FIGURE 4:**
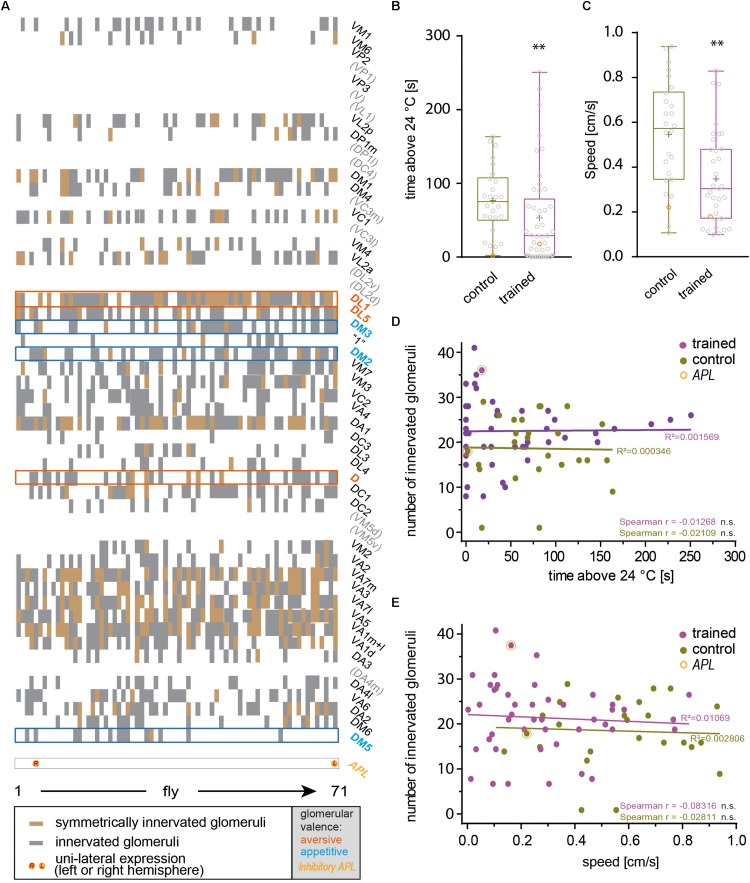
The total number of artificially activated glomeruli does not correlate with learned avoidance. **(A)** Glomeruli innervated by mCherry-dTRPA1-expressing OPNs were determined in 71 individual flies that received a 4 h induction of gene expression during development. In each fly a stochastic population of OPNs showed expression, either symmetrically between the two brain hemispheres or only on one hemisphere. Glomeruli that have been reported to mediate olfactory aversion or attraction are indicated in magenta or cyan, respectively. Twenty-six animals were trained by pairing thermogenetic activation with electric shocks, 45 animals served as controls and did not receive electric shocks. Flies showed a reduction in the time spent >24°C **(B)** and in locomotion speed **(C)** when data from all animals, irrespective of the valence the glomeruli innervated by mCherry-dTRPA1-expressing OPNs mediate, were taken into account. **(D,E)** The total number of innervated glomeruli did not correlate significantly (Spearman *R*: n.s.) with the locomotion speed or the time spent >24°C. Lines indicate calculated linear correlations with coefficients (*R*^2^) indicated (see Table 4 for details). n.s., *p* > 0.05; ^∗∗^*p* < 0.01.

The identity of OPNs expressing mCherry-dTRPA1 was largely stochastic and differed between the two brain hemispheres. A Runs test for each glomerulus and for each brain hemisphere independently confirmed that for most glomeruli included in the expression pattern of GH146-Gal4 mCherry*-*dTRPA1 expression occurred stochastically, with the exception of VL2a on theleft hemisphere and VA1ml on both hemispheres. In these two cases expression occurred more often than predicted for complete randomness (Table 3). The glomeruli innervated by the OPNs could be identified. We utilized this knowledge to test whether the identity of OPNs and their odor-specific input to the mushroom body has relevance to efficient learning. For example, a more symmetric, and therefore more unambiguous, mushroom body input could potentially be learned more efficiently; the actual number of active OPNs, and therefore the “intensity” of mushroom body input, or the innate behavioral valence of the odor signaled via the activity of distinct OPNs could potentially affect aversive associative learning. Alternatively, the function of the mushroom body might not depend on the actual source of the input. In this case, learning would not be expected to be influenced by the parameters indicated above. To distinguish among these alternatives, the thermogenetic learning experiment was repeated using 4 h of heat shock during development. Subsequently, the brains of the tested flies were removed from the head capsule, subjected to immunohistochemical staining, and the glomeruli that harbored OPNs expressing mCherry-dTRPA1 were identified. Figure 3 exemplifies how immunostaining against the active zone protein Bruchpilot (Wagh et al., 2006) was used to identify all glomeruli in comparison with stochastic mCherry fluorescence. It should be noted that some glomeruli were innervated by mCherry-dTRPA1-expressing OPNs symmetrically between the brain hemispheres, which were sometimes visible only in different confocal planes (e.g., glomerulus VC2 in Figure 3). A total of 71 flies were analyzed, 26 of which were subjected to the associated training procedure and 45 to control conditions, without electric shocks (Figure 4A). Trained animals spent significantly less time at temperatures >24°C (Figure 4B). Moreover, this effect was accompanied by an overall reduction in the speed of locomotion (Figure 4C). However, no correlation between the actual number of glomeruli innervated by the mCherry-dTRPA1-expressing OPNs and temperature preference or locomotion speed was detected in trained or control animals (Figures 4D,E). These data suggest that flies can associate this neuronal signal with an unconditioned stimulus provided that a threshold number of OPNs is reached (Figure 2F); learning efficiency as measured by memory expression was not dependent on the actual number of activated OPNs. However, thermogenetic activation of all neurons covered by the GH146-Gal4 line simultaneously with electric shocks did not lead to associative learning (Supplementary Figure 2), indicating that there is also an upper limit of how many OPNs can be activated to serve as neuronal correlate of a conditioned stimulus. Alternatively, the fact that the inhibitory anterior paired lateral (APL) neuron, which innervates calyx and lobes of the ipsilateral mushroom body, is included in the expression pattern of GH146-Gal4 (Wilson and Laurent, 2005) might prevent successful associative conditioning in this case. In fact, it has been reported that in aversive associative learning the APL neuron becomes inhibited (Zhou et al., 2019), which is precluded in our case by the thermogenetic activation. However, in those experiments that involved a heat-induced stochastic expression of mCherrydTRPA1- only 2 out of 71 tested flies showed unilateral expression in the APL neuron (Figure 4A).

**TABLE 3:** Stochastic expression of heat shock induced mCherry-dTRPA1 expression in GH146-Gal4 targeted olfactory projection neurons confirmed by a Runs test for individual glomeruli on each brain hemisphere.

Glomerulus		Left		Right	Glomerulus		Left		Right
VM1	h:0	*p* = 0.8929	h:0	*p* = 0.8605	VM5d	h:0	*p* = 0.7057	h:0	*p* = 1
VM6	h:0	*p* = 1	h:0	*p* = 1	VM5v	h:0	*p* = 1	h:0	*p* = 0.6976
VP2	h:0	*p* = 1	h:0	*p* = 1	VM2	h:0	*p* = 0.3264	h:0	*p* = 0.4987
VP1	h:0	*p* = 1	h:0	*p* = 1	VA2	h:0	*p* = 0.1127	h:0	*p* = 0.4064
VP3	h:0	*p* = 1	h:0	*p* = 1	VA7m	h:0	*p* = 0.3306	h:0	*p* = 0.2725
V	h:0	*p* = 1	h:0	*p* = 1	VA3	h:0	*p* = 0.6191	h:0	*p* = 0.5301
VL1	h:0	*p* = 0.5090	h:0	*p* = 0.5090	VA7I	h:0	*p* = 0.6308	h:0	*p* = 1
VL2p	h:0	*p* = 0.2527	h:0	*p* = 0.9194	VA5	h:0	*p* = 0.6526	h:0	*p* = 1
DP1I	h:0	*p* = 1	h:0	*p* = 1	**VA1ml	h:1	*p* = 0.0400	h:1	*p* = 0.0095
DP1m	h:0	*p* = 1	h:0	*p* = 1	VA1d	h:0	*p* = 0.6138	h:0	*p* = 0.7571
DC4	h:0	*p* = 1	h:0	*p* = 1	DA3	h:0	*p* = 0.6808	h:0	*p* = 0.6808
DM1	h:0	*p* = 0.8641	h:0	*p* = 0.3690	DA4m	h:0	*p* = 1	h:0	*p* = 1
DM4	h:0	*p* = 0.0618	h:0	*p* = 0.6013	DA4I	h:0	*p* = 1	h:0	*p* = 0.8928
VC3m	h:0	*p* = 1	h:0	*p* = 0.7057	VA6	h:0	*p* = 0.3732	h:0	*p* = 0.5525
VC1	h:0	*p* = 0.6036	h:0	*p* = 0.7994	DA2	h:0	*p* = 0.4988	h:0	*p* = 0.1956
VC3I	h:0	*p* = 1	h:0	*p* = 1	DM6	h:0	*p* = 0.7047	h:0	*p* = 0.8929
VM4	h:0	*p* = 0.1653	h:0	*p* = 1	DM5	h:0	*p* = 0.0930	h:0	*p* = 0.1800
* VL2a	h:1	*p* = 0.0197	h:0	*p* = 1	VC2	h:0	*p* = 0.9349	h:0	*p* = 0.5642
DL2v	h:0	*p* = 0.7057	h: 0	*p* = 1	VA4	h:0	*p* = 1	h:0	*p* = 0.2251
DL2d	h:0	*p* = 0.3111	h:0	*p* = 1	DA1	h:0	*p* = 0.2725	h:0	*p* = 1
DL1	h:0	*p* = 0.5301	h:0	*p* = 0.105	DC3	h:0	*p* = 0.3111	h:0	*p* = 1
DL5	h:0	*p* = 0.7756	h:0	*p* = 0.5124	DL3	h:0	*p* = 0.4064	h:0	*p* = 0.0991
DM3	h:0	*p* = 0.5383	h:0	*p* = 0.5176	DL4	h:0	*p* = 0.9225	h:0	*p* = 0.4973
“”1””,	h:0	*p* = 1	h:0	*p* = 0.7057	D	h:0	*p* = 0.1786	h:0	*p* = 0.7800
DM2	h:0	*p* = 0.6330	h:0	*p* = 1	DC1	h:0	*p* = 0.3336	h:0	*p* = 0.9347
VM7	h:0	*p* = 1	h:0	*p* = 0.9349	DC2	h:0	*p* = 0.9225	h:0	*p* = 1
VM3	h:0	*p* = 0.8721	h:0	*p* = 0.9614	(Figure 4D) Runs test on contingency of expression pattern with *h* = 0 indicating that the expression values of glomerulus are in random order at the default 5% significance level.				

n.s., *p* > 0.05; *p < 0.05.

**TABLE 4:** Correlations between innervation characteristics, and time spent > 24°C and speed in control and trained animals.

Correlation	Exp. group	*R*^2^	Spearman *r*	*p*-value		Figures
Number of innervated glomeruli vs. time > 24°C (s)	Trained	0.0001569	–0.01268	0.9341	n.s.	4D
	Control	0.000346	–0.02109	0.9186	n.s.	
Number of innervated glomeruli vs. speed (cm/s)	Trained	0.01069	–0.08316	0.5940	n.s.	4E
	Control	0.002806	–0.02811	0.8916	n.s.	
Number of innervated app. glomeruli vs. time > 24°C (s)	Trained	0.002525	–0.05034	0.7397	n.s.	5B
	Control	0.1036	0.3127	0.0814	n.s.	
Number of innervated avers. glomeruli vs. time > 24°C (s)	Trained	3.631 * e^–005^	–0.1277	0.3975	n.s.	5C
	Control	0.003145	0.06007	0.7440	n.s.	
Number of innervated app. glomeruli vs. speed (cm/s)	Trained	0.0002323	0.02763	0.8554	n.s.	5D
	Control	0.046	0.2016	0.2686	n.s.	
Number of innervated avers. glomeruli vs. speed (cm/s)	Trained	0.01958	–0.0819	0.5885	n.s.	5E
	Control	0.006542	–0.07814	0.6708	n.s.	
Symmetry vs. time > 24°C (cm/s)	Trained	0.002375	–0.02268	0.8824	n.s.	6A
	Control	0.02534	0.1566	0.4450	n.s.	
Symmetry vs. speed (cm/s)	Trained	0.00363	0.1006	0.5107	n.s.	6B
	Control	0.01826	0.1528	0.4562	n.s.	

### No Correlation Between the Valence Signaled by OPNs and Aversive Associative Learning

**FIGURE 5:**
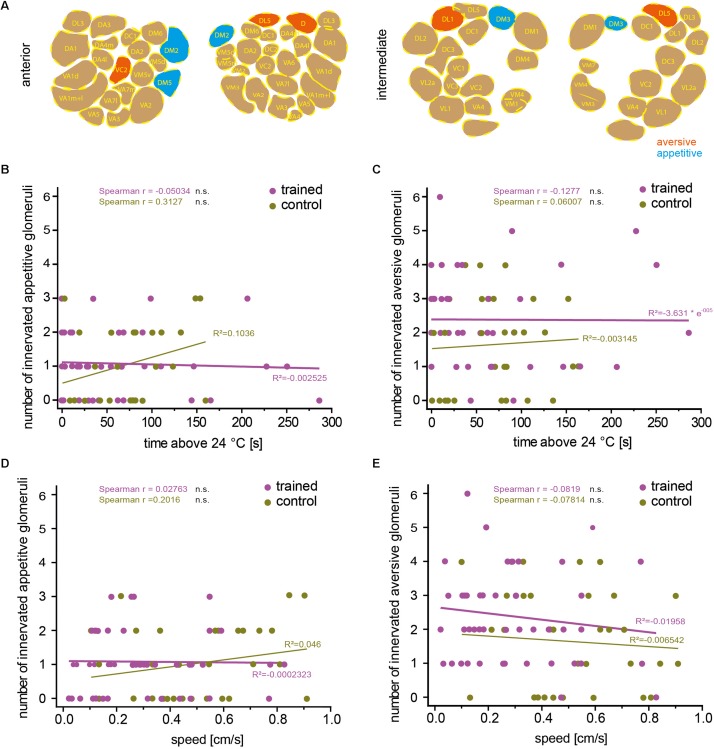
The number of attraction- or aversion-mediating glomeruli does not correlate with learned avoidance. **(A)** Example of one brain showing stochastic mCherry-dTRPA1 expression in attraction (green) or aversion-mediating (magenta) glomeruli in a symmetric or non-symmetric manner. The number of “appetitive” **(B)** or “aversive” **(C)** glomeruli did not correlate significantly (Spearman *R*: n.s.) with the time the trained animals spent >24°C, and **(D,E)** did not correlate significantly (Spearman *R*: n.s.) with the animals’ locomotion speed. Lines are calculated linear correlations with coefficients (*R*^2^) indicated (see Table 4 for details).

Odors can influence diverse behaviors such as feeding or oviposition and can therefore act as attractive or aversive cues. Highly stereotyped connectivity from the olfactory sensory neurons to the OPNs and similar stereotyped olfactory receptor expression has enabled researchers to correlate the activity of distinct neurons with attractive or aversive valence (Semmelhack and Wang, 2009; Knaden et al., 2012), and induce either attraction or aversion via artificial activation of distinct neurons (Bellmann et al., 2010). Knaden et al. (2012) found that the activity of OPNs is more distinctly indicative for the valence of the odor-evoked behavior compared with sensory neuron activity. The OPNs innervating glomeruli DM2, DM4, DM5 responded predominantly to attractive odorants, whereas those innervating glomeruli D, DL1, and DL5 responded to aversive odorants (Knaden et al., 2012). We asked whether aversive associative learning is affected in either direction if OPNs that are activated primarily by attractive or repulsive odorants express mCherry-dTRPA1 within the overall stochastic expression pattern. Glomeruli expressing mCherry-dTRPA1 in the 71 analyzed flies were determined (Figures 4A, 5A). None of the flies showed expression in the OPNs innervating DL2. However, the remaining valence-indicative glomeruli were included in the stochastic expression patterns. No correlation was observed between the number of attraction-mediating or repulsion-mediating glomeruli in either brain hemisphere and the duration of time that the trained animals spent >24°C (Figures 5B,C). There was also no correlation between the number of attractive or aversive glomeruli and the locomotion velocity of movement after training (Figures 5D,E). Thus, our analysis did not reveal any potential influence of the valence the OPNs signal and the efficiency of aversive associative learning or memory expression. This finding is perhaps not entirely surprising considering how learned information can override naïve information, e.g., through appetitive conditioning using innately aversive odorants.

### No Correlation Between Inter-Hemispheric Symmetry of OPN Activity and Aversive Associative Learning

**FIGURE 6:**
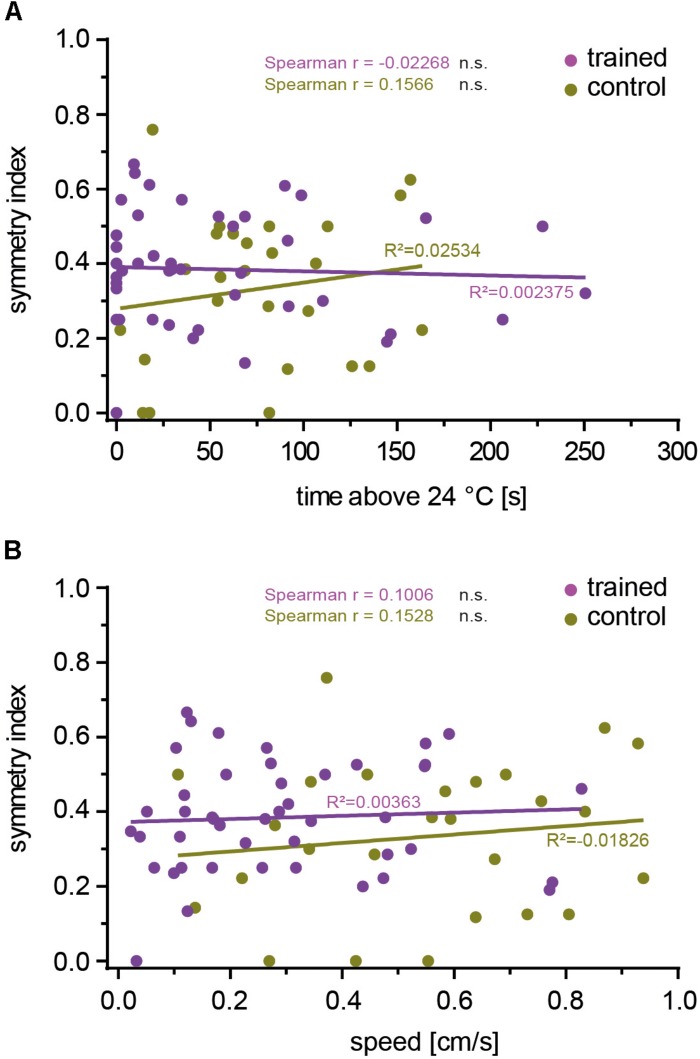
The inter-hemispheric symmetry between glomeruli innervated by mCherry-dTRPA1 does not affect learning. The degree of symmetry, as determined by a symmetry index (see the section “Materials and Methods”), was not significantly (Spearman *R*: n.s.) correlated with the time the flies spent >24°C after learning **(A)** or locomotion speed **(B)**, either after associative training or under control conditions (see Table 4 for details).

Our data did not indicate that the particular individuality of the OPNs that provide input to the mushroom body skews the efficiency of associative learning in either direction. Rather, it appeared that the mushroom body did not take the actual identity of OPN input into account. If so, the efficiency of associative learning should also be independent of whether OPNs are symmetrically activated between brain hemispheres or not. To test this hypothesis, we calculated a symmetry index (see the section “Materials and Methods”) that quantified the degree of inter-hemispheric symmetry between identified glomeruli innervated by mCherry-dTRPA1-expressing OPNs. Indeed, no correlation between the symmetry index and the time the flies spent >24°C or their locomotion speed was detected in either trained animals or controls (Figures 6A,B). This result suggests that learned avoidance behavior is independent of the degree of inter-hemispheric symmetry of glomerular activity.

## Discussion

The neuronal circuits that control an animal’s behavior are often highly stereotyped between individuals. Evolution has optimized these circuits to fulfil the requirements imposed by the ecology and life history of the species. For example, *Drosophila* has evolved mechanisms to detect the nutritious, fermenting fruits of particular plants and to be attracted to them. Similarly, mechanisms have evolved to avoid harmful substances. Thus, stereotyped gene expression and neuronal circuit wiring reflect innate ecological and behavioral programs. This is also reflected in stereotyped chemotopic maps, observable in the antennal lobes (Fiala et al., 2002; Wang et al., 2003), that result from fixed olfactory-receptor expression and hard-wired neuronal connectivity (Vosshall et al., 2000; Couto et al., 2005; Fishilevich and Vosshall, 2005). Such hard-wired and stereotyped connectivity is also found at higher-order brain regions, such as in the projection areas of OPNs in the lateral horn (Marin et al., 2002; Wong et al., 2002; Tanaka et al., 2004) or intrinsic lateral horn neurons (Strutz et al., 2014; Jeanne et al., 2018; Frechter et al., 2019). The innate behavioral valence of odorants is more reliably and distinctly represented by the combinatorial activity pattern of second-order OPNs compared with first-order olfactory sensory neurons (Knaden et al., 2012). This is in line with the stronger categorization of combinatorial odor representations in OPNs compared with those in sensory neurons (Niewalda et al., 2011). Interestingly, representation of the innate valence of an odor is segregated in different partitions of the lateral horn (Strutz et al., 2014; Seki et al., 2017), indicating that this hard-wired connectivity extends beyond the sensory periphery to the behavior-instructing neuropils.

By contrast, learning is a mechanism that allows organisms to deal with environmental unpredictability. As a human commensal (Keller, 2007), fruit flies have to adapt to environments that differ from their ancestral African habitat (Mansourian et al., 2018). Therefore, the ability to learn appears essential for their survival. The well-documented random, variable, non-stereotyped connectivity between OPNs and Kenyon cells at the calyx of the mushroom body (Murthy et al., 2008; Caron et al., 2013) is thought to reflect this environmental unpredictability (e.g., Luo et al., 2010), despite some degree of spatially determined projections of OPN axons in the calyx (Lin et al., 2007; Christiansen et al., 2011). Key factors that distinguish the mushroom bodies from other neuropils, like the lateral horn, include a relatively high number of uniform intrinsic neurons (Kenyon cells) that lack apparent individual genetic identities, and the highly selective responsiveness of a very few of those Kenyon cells (∼5%) out of a large number (∼2000) (Honegger et al., 2011; Gruntman and Turner, 2013) (sparse code). The principle of randomly generated, sparsely distributed neuronal activity as a favorable memory store was formally described in 1988 (Kanerva, 1988), and in a diversity of neuronal circuits this principle has been found to be implemented, including the cerebellum, the piriform cortex, and the mushroom body (Babadi and Sompolinsky, 2014; Litwin-Kumar et al., 2017). This suggests that the exact identity of the neurons that encode a learned stimulus is irrelevant for the functionality of the circuit. Indeed, arbitrarily activated, stochastic patterns of piriform cortex cells can be learned by mice as being either aversive or attractive (Choi et al., 2011). Similarly, *Drosophila* can learn to behaviorally avoid activation of a stochastic, arbitrary pattern of Kenyon cell activity that has been temporally paired with a punishing electric shock (Vasmer et al., 2014). However, this suggests that the exact circuit input itself is not a deterministic factor for the Kenyon cells to be used as a learnable pattern. Rather, Kenyon cells can use any input pattern as template to be associated with a rewarding or punishing stimulus. This does not necessarily implicate that the sensory input of the insect mushroom body is anatomically or functionally chaotic. In fact, input into the calyces of mushroom bodies are often highly structured and segregated according to the sensory modality they convey. However, the mushroom body can learn any input pattern, irrespective of the exact identity of the OPNs, the exact odor and the odor valence it signals, or the symmetry between the two hemispheres. The data presented here are consistent with this idea.

The animals’ learning-dependent avoidance to reactivate OPNs was observed only within the first 5 min of the test situation, and not at later time points. This time-dependent decrease in learned avoidance might perhaps be due to an intrinsic adaptation of OPNs to continuous excitation (Cafaro, 2016; discussion in: Martelli and Fiala, 2019). This is in contrast to the slowly developing occurrence of learned avoidance thermogenetically induced in Kenyon cells (Vasmer et al., 2014), pointing toward different physiological properties of these cells. It must also be noted that when the animals move along the test arena to temperatures >25°C, excitation of OPNs expressing mCherry-dTRPA1 is likely to increase in dependence of the ambient temperature. However, this potential change in excitation does probably not resemble changes in real odor concentration, because the identity of excited OPNs is determined by the expression of mCherry-*d*TRPA1. These potential variations in excitation do likely not result in different combinatorial activities of OPNs or a recruitment of active glomeruli, as it is the case for increasing odor concentrations (Wang et al., 2003). It might be interesting to see in the future whether the principle of using stochastic input patterns into the mushroom body to override innate behavioral tendencies through learning is also true for appetitive conditioning using sugar reward as unconditioned stimulus.

It should be noted that most of the animals tested in this study showed expression of the thermogenetic actuator mCherry-dTRPA1 in both aversive and attractive glomeruli. The net effect of the combined activity of both remains unclear. Moreover, the probability of achieving expression in a large proportion of symmetric glomeruli between the hemispheres is low, leaving open the possibility that an exact symmetric activation between the hemispheres might have a more profound effect on learnability. However, we can conclude that the animals have the ability to learn stochastic and arbitrarily activated ensembles of OPN activity and to subsequently avoid their re-activation. When a real odor stimulus is learned, the innate valence represented by the combinatorial OPN pattern and signaled to the lateral horn (Strutz et al., 2014) has to be integrated with the learned information to induce an appropriate behavior. This cross-talk between the mushroom body and the lateral horn has been characterized (Dolan et al., 2018).

In other insects with more elaborate mushroom bodies and often much more complex behavior, such as eusocial hymenoptera (e.g., honey bees or ants), butterflies, or cockroaches, large parts of the mushroom body calyces receive not only olfactory, but also massive multimodal, visual, and mechanosensory input (Mobbs, 1982; Gronenberg and Hölldobler, 1999; Strausfeld and Li, 1999; Gronenberg, 2001; Ehmer and Gronenberg, 2002; Strausfeld, 2002; Paulk and Gronenberg, 2008; Kinoshita et al., 2015). Anatomically, the different sensory input fibers targeting the calyces are not randomly organized, but show a high degree of orderly structure. The less-complex mushroom body of *Drosophila* is dominated by olfactory input; however, afferent sensory fibers providing information about temperature (Frank et al., 2015) or visual input (Vogt et al., 2016; Yagi et al., 2016) also exist. It might be interesting to investigate in the future whether also for the representation of sensory modalities other than olfactory ones and also in insects with more complex mushroom bodies the diverse “input patterns” are integrated by Kenyon cells in a stochastic manner or, alternatively, whether in these cases exact topographical representations (e.g., retinotopic activity patterns) or stereotypic labeled lines are of importance for the behavioral functions of mushroom bodies, such as associative learning.

## Data Availability Statement

The raw data supporting the conclusions of this article will be made available by the authors, without undue reservation, to any qualified researcher.

## Author Contributions

AF conceived the project and wrote the manuscript. AF and TR supervised the work. TR, CW, and PF performed the experiments and analyzed the data.

## Conflict of Interest

The authors declare that the research was conducted in the absence of any commercial or financial relationships that could be construed as a potential conflict of interest.
