# Supplementary figures and images for: Stochastic and Arbitrarily Generated Input Patterns to the Mushroom Bodies Can Serve as Conditioned Stimuli in Drosophila
Source: Front Physiol. 2020 Feb 11;11:53. doi: 10.3389/fphys.2020.00053 (PMC7027390; doi:10.3389/fphys.2020.00053)

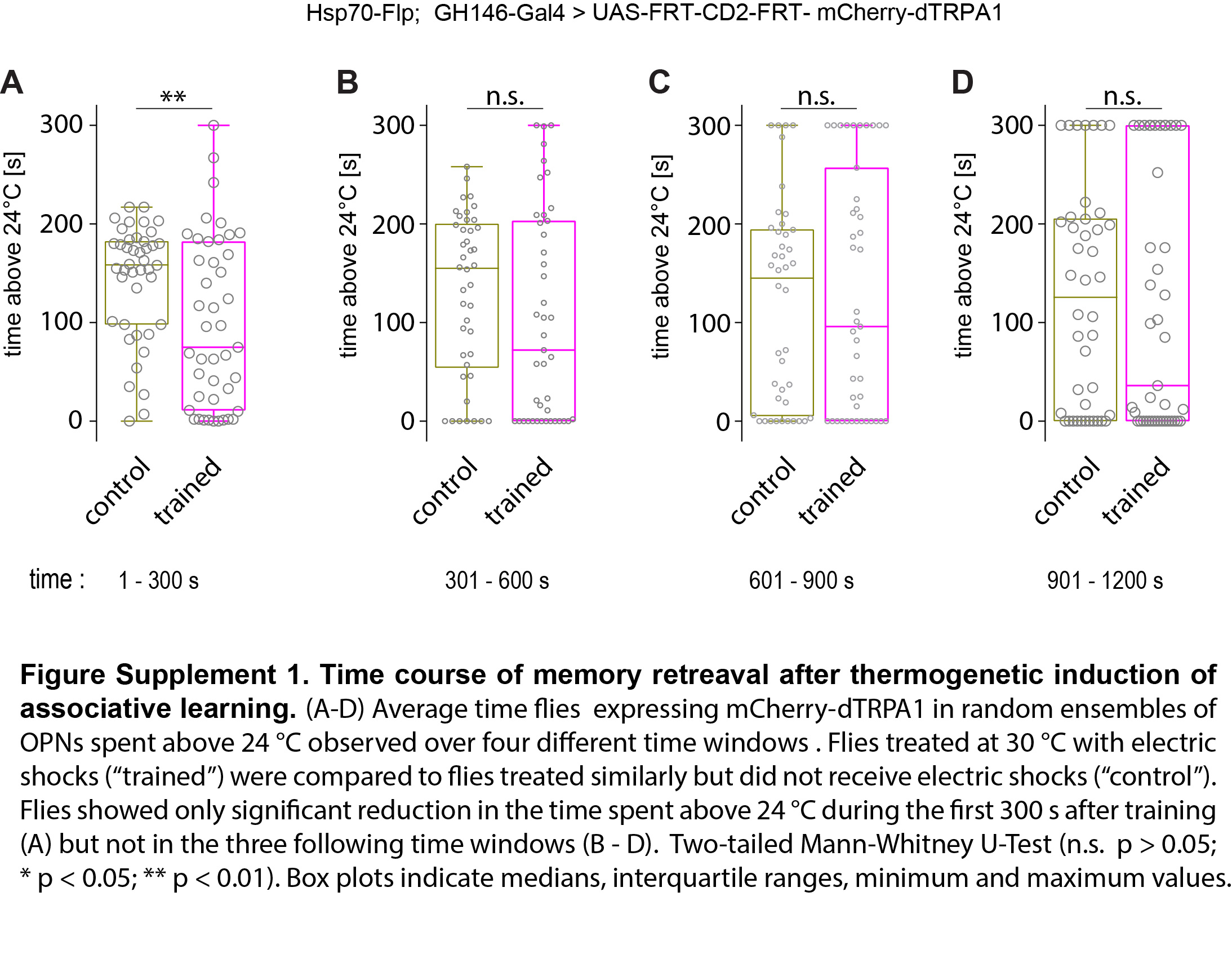

Supplement: Supplementary file 1 [file Image_1.jpeg]

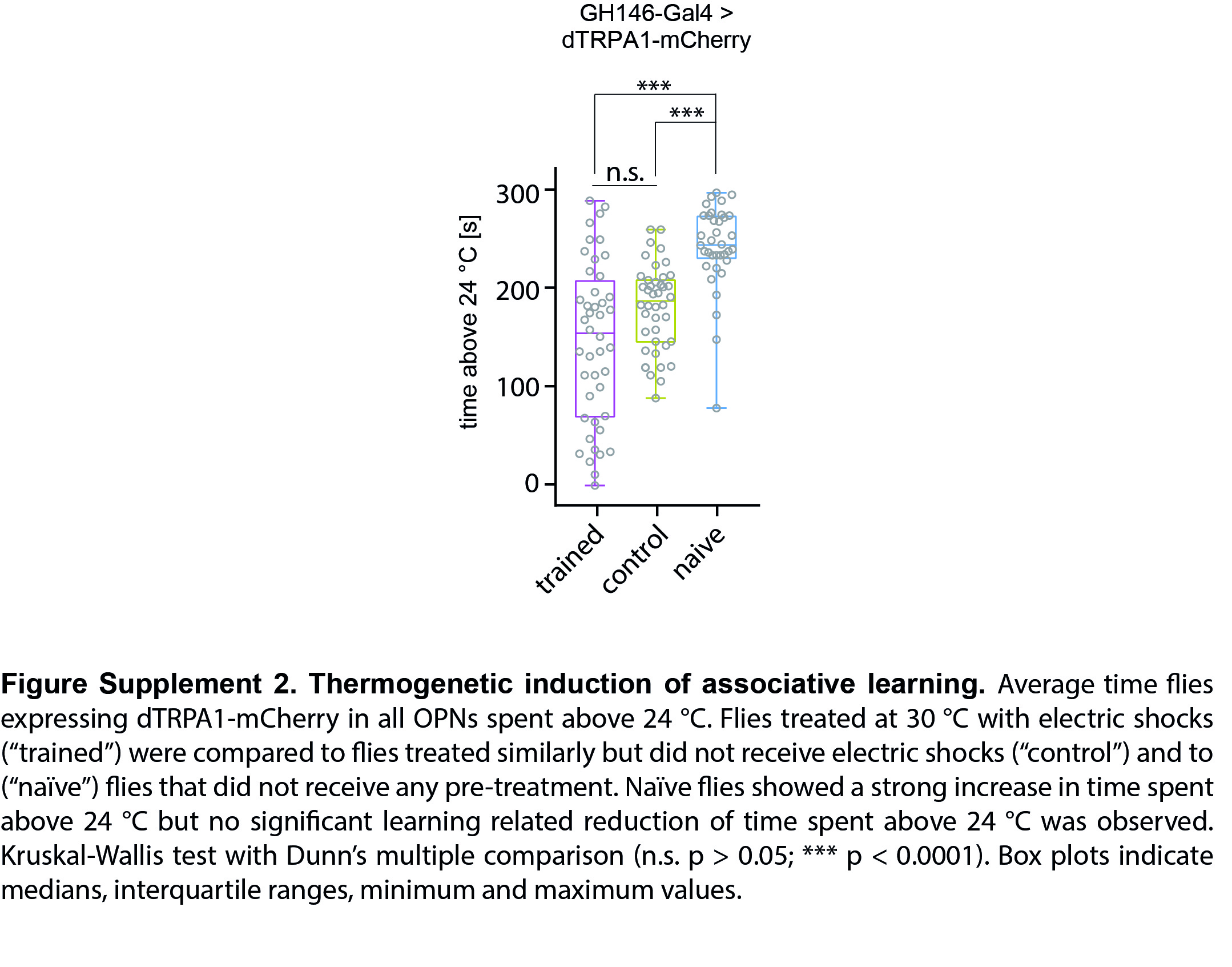

Supplement: Supplementary file 2 [file Image_2.jpeg]
